# Clinical outcome and prognostic factors in recurrent oral squamous cell carcinoma after primary surgical treatment: a retrospective study

**DOI:** 10.1007/s00784-021-04186-y

**Published:** 2021-09-28

**Authors:** Sven Zittel, Julius Moratin, Dominik Horn, Karl Metzger, Oliver Ristow, Michael Engel, Jan Mrosek, Kolja Freier, Jürgen Hoffmann, Christian Freudlsperger

**Affiliations:** 1 Department of Oral and Cranio-Maxillofacial Surgery, University Hospital Heidelberg, Im Neuenheimer Feld 400, D-69120 Heidelberg, Germany; 2 Saarland University Hospital, Department of Oral and Cranio-Maxillofacial Surgery, Kirrberger Straße, D-66424 Homburg, Germany

**Keywords:** Recurrent oral squamous cell carcinoma, Salvage surgery, Oral cancer, Ablative surgery, Disease-free interval

## Abstract

**Objectives:**

Survival for patients with recurrent oral squamous cell carcinoma is usually poor, and the most effective treatment has not yet been clearly defined. The present study evaluates the outcome in radiotherapy-naïve patients after recurrence of oral squamous cell carcinoma with respect to different treatment modalities including surgery, radiation, chemoradiation, and palliative treatment.

**Patients and methods:**

In this retrospective study, we included all patients with primary oral squamous cell carcinoma who received exclusively surgical therapy between 2010 and 2020 and who suffered from locoregional recurrence in their follow-up. Patients with previous adjuvant therapy were excluded from this protocol. Clinical and pathological parameters were collected and statistically evaluated. Survival analysis was performed according to Kaplan–Meier. The primary endpoints were overall and progression-free survival in dependance of treatment strategy for recurrent tumors.

**Results:**

Out of a total of 538 patients with surgically treated primary oral squamous cell carcinoma, 76 patients met the inclusion criteria. The mean follow-up was 38 ± 32 months. Patients who received surgically based therapy had a significantly better outcome in terms of disease-free survival (DFS) and overall survival (OS) (DFS *p* < 0.001; OS *p* < 0.001). The presence of regional metastases and a short disease-free interval (DFI) between primary and recurrent cancer were significant predictors for adverse outcomes (DFI *p* < 0.001).

**Conclusion:**

We recommend primary surgical therapy for radiotherapy-naïve patients with recurrent oral squamous cell carcinoma, supplemented by risk-adapted adjuvant therapy.

**Clinical relevance:**

Surgical therapy continues to play a central role in the treatment of radiotherapy-naïve patients with recurrent oral squamous cell carcinoma.

## Introduction

Head and neck squamous cell carcinoma (HNSCC) is one of the most common cancer entities worldwide and belongs to the heterogeneous group of head and neck cancer [1–3]. Within HNSCC, oral squamous cell carcinoma (OSCC) represents one of the most important subsites [4]. Surgical therapy, i.e., resection of the malignancy combined with elective neck dissection (END), is the most common modality for the primary treatment of OSCC. It is accompanied by adjuvant radiotherapy or radiochemotherapy according to the histopathological staging and the presence of risk factors [5, 6]. Despite intense research on molecular and clinical level, OSCC recurrence rates and overall survival rates have barely changed for decades [7, 8]. Even in early-stage disease, recurrences occur in 10–25% [6, 9]. In advanced-staged disease, tumor recurrence is a frequently observed problem and occurs in approximately 40–60% of cases [10–13] resulting in a poor overall survival [14]. Primary tumor stage and histopathological grading seem to be mainly predictive for relapse [15].

The term of salvage surgery is not used consistently throughout the available literature. In the surgical treatment of a recurrent OSCC, a distinction is needed between heavily pretreated patients who had previous adjuvant radio(chemo)therapy and those who had only received surgery as their primary treatment. Patients with a history of multi-modal therapy are more challenging to treat and have a higher risk of early relapse [16]. Patients without previous radiotherapy have shown to have a better outcome after tumor recurrence [17].

Treatment options for recurrent OSCC are becoming more patient-specific and a few studies have been conducted to evaluate treatment regimens and outcome rates of HNSCC [18, 19]. Even though the introduction of immune-checkpoint-inhibition has revolutionized the treatment of OSCC, it is still reserved for the palliative setting [20, 21].

Currently, curative treatment of recurrent OSCC includes surgery, radiotherapy, and chemoradiation [18]. However, decision-making in recurrent OSCC is not based on evidence, as literature is lacking data on outcome rates and chosen treatment strategy [19]. Therefore, the purpose of this study was to determine which treatment regimen is most effective for recurrent radiotherapy-naïve OSCC and what specific risk factors might influence decision-making. For a more straightforward description of patients which have not been treated with radiotherapy before, we use the term “radiotherapy-naïve” in the following.

## Patients and methods

### Data collection

This retrospective study includes all patients with recurrent OSCC who have only received surgical treatment of their primary tumor without any adjuvant treatment in the period between 2010 and 2020 at the Department of Oral and Cranio-Maxillofacial Surgery of the Heidelberg University Hospital. Exclusion criteria were a history of radio- or chemo-radiotherapy as primary or adjuvant treatment of the primary OSCC. The study was approved by the local ethics advisory board of the Heidelberg University (Ethic vote S-183/2015). Their consultation is based on the valid professional code of conduct and the relevant declaration of the World Medical Association of Helsinki in the current version. Written informed consent was provided by all patients. Clinical data were collected from the electronic patient records.

### Treatment

Treatment planning of the index tumor followed the German national guidelines for OSCC after tumor staging with computed tomography (CT) of head, neck, and chest and histopathological confirmation of the diagnosis [22]. All patients were surgically treated according to this guideline. If histopathological findings showed close resection margins, lymph node metastases, or accumulations of histopathological risk factors (perineural invasion, lymph and blood vessel invasion), the patients received adjuvant radiotherapy or platinum-based radiochemotherapy. These patients were then excluded from this study. Clinical, histopathological, and follow-up data were collected and assessed with SAP Patient Management research software (SAP, Walldorf, Germany). The following parameters were collected and assessed: age, sex, tumor stage, nodus stage, UICC stage, margins, tumor grading, disease-free interval and tumor localization, further treatment. Further recurrence-specific parameters were collected as follows: type of recurrence, recurrent tumor stage, recurrent tumor localization, recurrent nodus stage, recurrent UICC stage, treatment of the recurrence, type of used reconstruction, occurrence of a second recurrence, and the interval from first to second recurrence. “Disease-free interval” was defined as the time between primary tumor disease and recurrence. “Disease-free interval after first recurrence” is the time from first to second relapse. For patients showing a locoregional relapse, surgical therapy always included oncological tumor resection with corresponding safety margins. Bilateral neck dissection of the levels I–III was (re-)performed or completed, respectively. Soft tissue reconstruction was performed in all patients using microvascular flaps or local flaps. If, following the operation, the tumor was classified as advanced disease, an adjuvant treatment was initiated. Patients who did reject surgery or suffered from an extensive and inoperable disease received definitive radiotherapy or palliative treatment depending on the patient’s case. All patients were integrated into a systematic recall. The follow-up procedure consisted of a clinical examination every 4 weeks in the first year and every 3–6 months from the second to the fifth year combined with a CT scan and sonography of the head and neck every 3 to 6 months.

### Statistics

The statistical evaluation was performed using Microsoft Excel 2013 (Microsoft, Redmond, WA, USA), SPSS Statistics version 25 (IBM, Armonk, NY, USA) and the statistical software R version 4.0.3. Apart from R’s base functionality, the following packages were used: survminer. Demographic and clinical data were collected and descriptively analyzed. Survival rates were analyzed using the Kaplan–Meier method. Log-rank testing was used to estimate the differences between the groups. Multivariate testing using Cox regression analyses was performed to determine the prognostic value of different factors with relevant co-variates. A ROC analysis (ROC (receiver operating characteristic)) was performed to determine a possible cutoff value. A *p*-value of 0.05 or less was considered to indicate statistical significance.

## Results

### Basic patient characteristics and first-line therapy

**Fig. 1:**
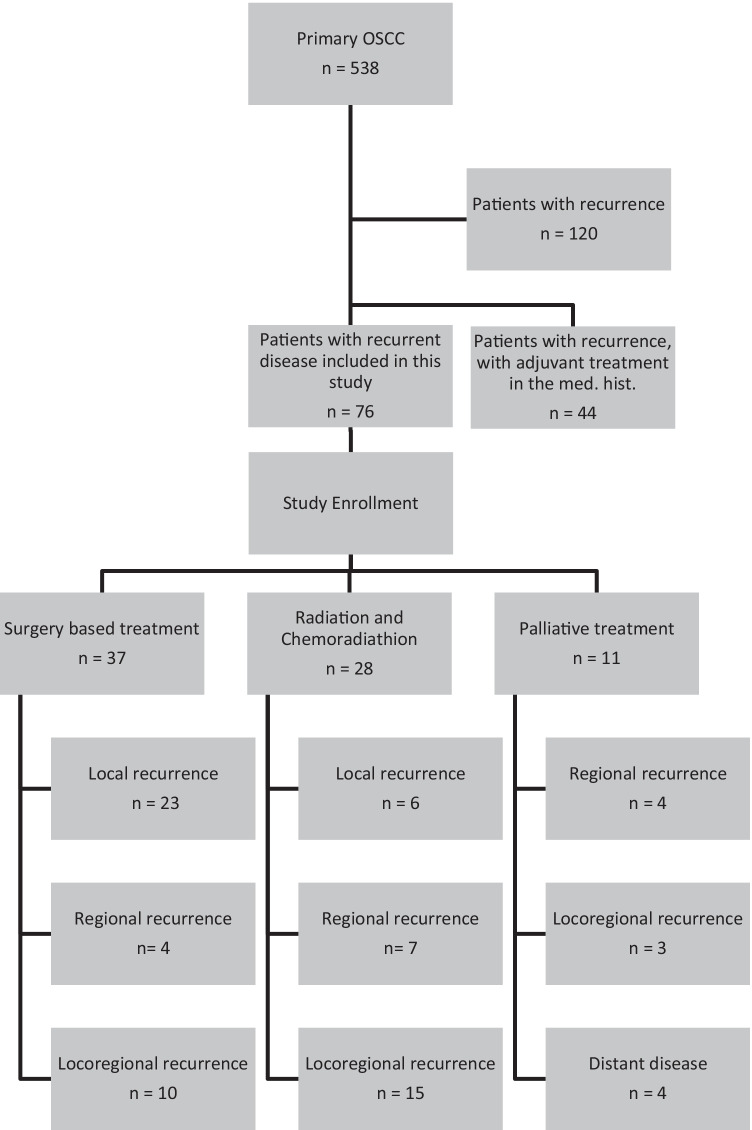
Cohort description in accordance to the chosen treatment and type of recurrence

We identified 538 patients with a primary OSCC who received primary surgical treatment in our department from 2010 to 2020. We observed a total of 120 (22.3%) patients experiencing a tumor recurrence during follow-up. Out of these 120 patients, we identified 76 (14.1%) patients who had previously only been treated surgically and were subsequently included in this analysis. All of the included 76 patients did not have any adjuvant treatment in their medical history (Fig. 1).

The mean patient age of the patients was 64.3 $\pm$ 12.3 years (range 31–88). The cohort consisted of 34 (44.7%) women and 42 (55.3%) men. The mean follow-up time was 38 $\pm$ 32 months. Thirty-one (40.3%) patients included in this study had an indication for adjuvant therapy for the index tumor due to histopathological parameters but either rejected adjuvant treatment of their index tumor or were not eligible due to general condition. Patients and tumor characteristics of the index OSCC are summarized in Table 1.

**Table 1:** Patient and tumor characteristics of the index OSCC

Parameter	Number of cases (%)
Age	
$\le$65	33 (43.4)
$>$65	43 (56.6)
Sex	
Female	34 (44.7)
Male	42 (55.3)
pT	
T1	33 (43.4)
T2	20 (26.8)
T3	5 (6.6)
T4	18 (23.7)
Tumor localization	
Tongue	16 (21.1)
Buccal mucosa	6 (7.9)
Tongue base	1 (1.3)
Floor of the mouth	21 (27.6)
Alveolar process	19 (25.0)
Lower lip	1 (1.3)
Maxilla	8 (10.5)
Soft plate	4 (5.3)
pN	
pN0	59 (77.6)
pN +	17 (22.4)
UICC stage	
I	31 (40.8)
II	14 (18.4)
III	09 (11.8)
IV	22 (28.9)
R-status	
R0	72 (97.3)
R1	2 (2.7)
Grading	
1	9 (11.8)
2	52 (68.4)
3	8 (10.5)
Missing	7 (9.2)
Disease-free interval	
$\le$12 months	42 (55.3)
$>$12 months	34 (44.7)
Adjuvant treatment	
Yes	0 (0)
No	76 (100)

*pT*, pathological tumor stage; *pN*, pathological lymph node stage; *pN0*, no lymph node metastasis after Neck dissection; *pN* + , lymph node metastasis after Neck dissection; *R-status*, margin status; *R0*, clear margin; *R1*, microscopic residual tumor

### Survival analysis in accordance to the index tumor

**Fig. 2:**
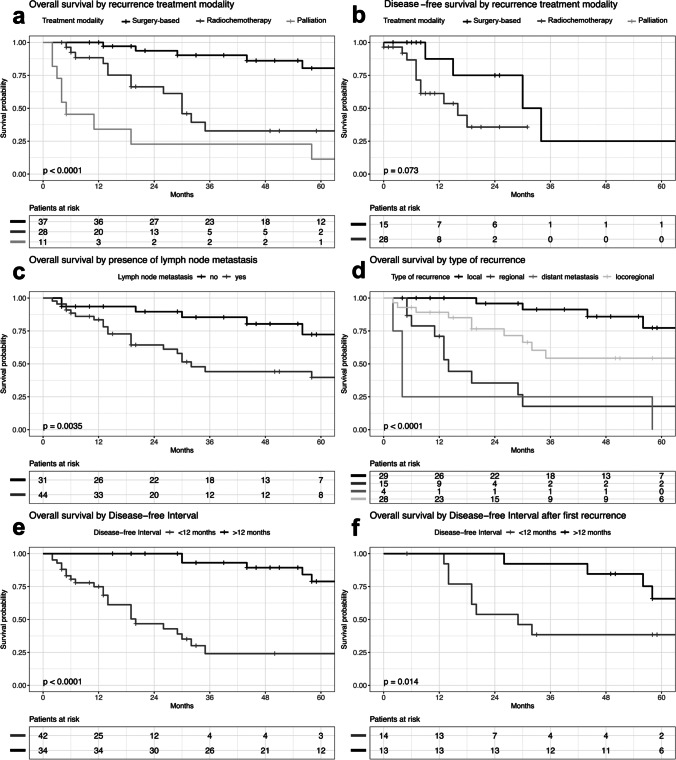
Analysis of overall survival and disease-free survival as a function of various parameters

The survival analysis in dependance of the index tumor showed a significantly poorer overall survival in patients with initial high UICC stage (stages 3 and 4) compared to patients with lower UICC stage (stages 1 and 2) (*p* < 0.001). The initial tumor stage (T) and the resection margin (R) did not impact survival significantly (pT: *p* = 0.456; R: *p* = 0.207) (Table 2). Furthermore, there was significant association of worse outcome in overall survival with the presence of lymph node metastases (*p* < 0.001) (Table 2). Additionally, groups were built in dependance of the period of time between the therapy of the index tumor and tumor recurrence. Here, we found a significant trend towards worse overall survival in patients with disease-free interval of < 12 months (*p* < 0.001) (Fig. 2e; Table 2). A ROC analysis showed a suitable value at 11.5 months based on our data. This value corresponds to our proposed cutoff value at 12 months. The area under the curve was 0.68, sensitivity was 0.69, and specificity was 0.66.

**Table 2:** Univariate Analysis of tumor characteristics of the index OSCC

Variable	*N* (%)	5-year OS probability in %	*p-*value
pT			
T1	33 (43.4)	54.8	0.456
T2	20 (26.3)	60.0	
T3	5 (6.6)	26.7	
T4	18 (23.7)	60.3	
pN			
pN0	59 (77.6)	65.6	< *0.001*
pN +	17 (22.4)	-	
R-status			
R0	72 (97.3)	52.4	0.207
R1	2 (2.7)	-	
UICC stage			
I and II	45 (59.2)	67.5	< *0.001*
III and IV	31 (40.8)	25.9	
Disease-free interval			
$\le$12 months	42	24.1	< *0.001*
$>$12 months	34	78.9	

Values set in italics marc significant values

*pT*, pathological tumor stage; *pN*, pathological lymph node stage; *pN0*, no lymph node metastasis after Neck dissection; *pN* + , lymph node metastasis after Neck dissection; *R-status*, margin status; *R0*, clear margin (> 0.5 mm); *R1*, microscopic residual tumor

### Characteristics of recurrences

In our cohort, 29 (38.2%) patients suffered from local recurrence, 15 (19.7%) patients suffered from regional recurrence, 28 (36.8%) patients suffered from locoregional recurrence, and 4 (5.3%) patients showed distant disease. The different localizations were statistically significant in our analysis (*p* < 0.001, Fig. 2d). As earlier mentioned, Fig. 1 gives an overview over patients, types of recurrent disease, and treatment modalities. Further relapse-specific parameters are listed in Table 3.

**Table 3:** Tumor and treatment characteristics of the recurrent OSCC with univariate survival analysis

Variable	*N* (%)	5-year OS probability in %	*p* value
Type of recurrence			
Local recurrence	55 (72.4)	69.7	< *0.001*
Regional recurrence	17 (22.4)	14.9	
Distant disease	4 (5.3)	0.0	
Recurrent T stage (rT)			
rT1	17 (22.4)	93.8	*0.021*
rT2	12 (15.8)	67.3	
rT3	0 (0.0)	-	
rT4	26 (34.2)	53.1	
No rT	21 (27.6)	11.9	
Recurrent tumor localization			
Tongue	7 (9.2)	*	0.276
Buccal mucosa	4 (5.3)		
Tongue base	3 (3.9)		
Floor of the mouth	13 (17.1)		
Alveolar process	15 (19.7)		
Maxilla	3 (3.9)		
Soft plate	2 (2.9)		
Cervical	17 (22.4)		
Distant	4 (5.3)		
Missing	8 (10.5)		
Recurrent N stage			
rN 0	31 (40.8)	72.3	*0.003*
rN +	44 (57.9)	39.7	
Missing	1 (1.3)		
Recurrent UICC stage			
I	15 (19.7)	92.9	< *0.001*
II	8 (10.5)	62.5	
III	6 (7.9)	80.0	
IV	47 (61.8)	31.7	
Treatment of the recurrent disease			
Surgery-based	37 (48.7)	80.4	< *0.001*
Chemoradiation only	28 (36.8)	32.8	
Palliative therapy	11 (14.5)	11.4	
Reconstruction surgical treated patients			
Local flap	6 (16.2)	60.0	< *0.001*
Free flap	30 (81.1)	87.8	
Pedicled flap	1 (2.7)	0.0	
2. Recurrence after treatment			
Yes	27 (35.5)	51.9	*0.760*
No	49 (64.5)	58.8	
Disease-free interval after first recurrent			
$\le$12 months	14 (51.9)	38.5	*0.014*
$>$12 months	13 (48.1)	65.8	

Values set in italics marc significant values

^*^Due to small subgroups, a closer analysis is not reasonable

*rT*, tumor stage of the recurrence; *rN*, lymph node stage of the recurrence; *rN0*, no lymph node metastasis of the recurrence; *pN* + , lymph node metastasis of the recurrence

Subgroups were formed for further analyses. The subgroup “surgery-based treatment” contains all patients receiving sole surgery or surgery plus adjuvant treatment. The subgroup “radiation” includes all patients receiving radio- or radiochemotherapy with curative intent. The subgroup “palliative treatment” includes all remaining patients.

The recurrences were treated as follows: 37 (48.7%) patients received a surgery-based treatment, 28 (36.8%) patients only received radio- or radiochemotherapy, and 11(14.5%) patients received a palliative treatment with chemotherapy and/or best supportive care.

### Survival analysis of the recurrent disease

The survival analyses in dependance of recurrence-specific parameters are shown in Table 3. As described, the type of recurrence was a statistically significant parameter in terms of overall survival with a favorable survival for local recurrence over regional recurrence and distant disease (*p* < 0.001) (Fig. 2d). During the further evaluation of the recurrence-specific parameters, we observed that recurrent UICC stage, the existence of lymph node metastases, and disease-free interval after primary treatment were statistically significant predictors for overall survival (OS rUICC: *p* < 0.001; OS rN: *p* = 0.003, Fig. 2c; OS rDFI: *p* = 0.014 Fig. 2f; Table 3). Overall survival and progression-free survival were mainly affected by the therapy received. Here, we observed a significant difference in overall survival and a trend in progression-free survival with respect to the selected modality (OS *p* < 0.001; PFS *p* = 0.073) (Fig. 2a, b).

In the multivariate Cox regression proportional hazards analysis, we identified the parameters “disease-free interval” and “age” were independent prognostic factors for overall survival in patients with recurrent OSCC (disease-free interval *p* < 0.001; age *p* 0.047, Table 4). “Recurrent UICC stage” was not an independent prognostic parameter for overall survival (*p* 0.066). Our data show stage-dependent therapy, with early stages more likely to require surgery (plus adjuvant therapy if necessary) and advanced stages more likely to require radiation or palliative therapy (Table 5).

**Table 4:** Multivariate Cox regression analysis of overall survival in the cohort suffering from recurrent OSCC

Parameter	HR (95% CI)	*p*-value
Recurrent UICC stage (I, II vs. III, IV)	4.337 (0.906–20.764)	*0.066*
Disease-free interval ($\le$12 months vs. > 12 months)	0.140 (0.055–0.352)	< *0.001*
Recurrence N stage (N0 vs. N +)	1.370 (0.385–4.878)	0.627
Age ($\le$65 years vs. > 65 years)	2.287 (1.011–5.175)	*0.047*

Values set in italics marc significant values

*HR*, hazard ratio; *CI*, confidence interval; *Recurrent UICC* Stage, UICC Staging of the recurrent tumor; *Recurrence N-stage*, Recurrent lymph node stage

**Table 5:** Distribution of the chosen treatment modalities in accordance to UICC stage

Recurrent UICC stage	Salvage surgery only	Salvage surgery with radiation	Radiation only	Chemo	Best supportive care
I	14	1	-	-	-
II	3	3	2	-	-
III	2	1	3	-	-
IV	3	10	23	3	8

## Discussion

The present study is aimed to evaluate the clinical outcome of a surgery-based therapy in radiotherapy-naïve patients with recurrent OSCC.

Recurrences are a frequently observed phenomenon and are associated with poor survival [14]. Various factors have been identified to have an influence on the occurrence of recurrent tumors and subsequently on the patient’s survival [15, 23, 24]. However, when it comes to therapeutic strategies, no uniform path has yet been defined as literature is lacking evidence.

### Effect of index tumor characteristics

When investigating recurrences and their therapy, primary disease and primary therapy should be considered. Our analysis shows that a high primary tumor stage is associated with a poor disease-free survival and a poor overall survival. A poor disease-free survival and a poor overall survival is also correlated with histopathologic risk factors, such as the existence of lymph node metastases. The existence and the amount of lymph node metastases are helpful parameters to assess the aggressivity of the tumor [23, 25]. In advanced-stage OSCC treatment guidelines recommend adjuvant therapy for improved disease-free and overall survival [26]. Unsurprisingly, positive lymph node status and advanced tumor stage of the index tumor were significant prognostic factors in our cohort. Other publications presented similar findings and could underline that a high stage of the primary tumor is correlated with a poor outcome and a higher risk for developing tumor recurrence [13, 27]. Since recurrence rates in early-stage OSCC are still around 10–25% and recurrence has a significant impact on survival, the primary therapy should aim to minimize the risk of recurrence. In addition to the radical removal of the tumor, therapy should include a thorough neck dissection [6, 28].

Despite the common understanding that margin status is a relevant and independent parameter for disease-free survival, we could not show such an effect with our cohort. This may be due to an underrepresentation of patients with positive margins in our cohort. Hosni et al. described that positive resection margin status is correlated with early recurrence [24]. Furthermore, there the association between clinicopathological parameters as grading and the time of recurrence is already shown [15]. According to Weckx et al., we could also find a correlation between histopathological risk factors and the rate of recurrence. Nevertheless, we could not show a relevant prognostic impact of tumor grading on overall survival.

Location of the index tumor and the type of recurrence were significant prognostic parameters in our cohort. The prognostic significance of the location of the primary and the recurrent tumor has also been shown by Ganan et al. Patients with local relapse had a significantly better outcome in overall survival than patients with regional recurrence. These findings are consistent with the results of other studies [29, 30].

The disease-free interval also seems to have an important effect on the overall survival of patients with recurrent OSCC. We observed a significant correlation between a short disease-free survival with presentation of recurrence within 12 months. We chose 12 months because it turns out to be a suitable time in the context of a ROC analysis. In addition, a cutoff at 12 months was very divisible in terms of group size and observed case numbers. However, it is important to point out the modest significance of the results, especially in view of the small number of cases. Other working groups chose different time periods to define the favorable disease-free interval. However, no clear time frame could be defined in the literature, so we oriented ourselves to decisions of other working groups and incorporated the characteristics of our cohort [15, 18, 27, 30]. This shows that there is still no agreement on a suitable cutoff. Even though other study groups are pointing out that the timing of recurrence has a strong effect on the outcome, the timing of recurrence is still not considered in the classification of relapses [15, 29–31]. In our study, we were able to show that the disease-free interval is one of the most relevant measures of overall survival in patients with recurrent oral squamous cell carcinoma. Thus, if the time to recurrence is assumed to be a prognostically relevant parameter, this should also be considered when deciding on the adjuvant modality in low staged recurrence. One could therefore conclude that more radical therapies should be taken into strong consideration, especially for early recurrence.

### Recurrence characteristics

In our univariate survival analysis of the recurrent tumors, we could show that there are relevant factors. Especially, the classical parameters such as the extent of the recurrent tumor and lymph node involvement were decisive. These patients turned out to have a poor outcome regarding overall survival. This, in turn, has been described in several publications and is therefore consistent with our observations [13, 25, 29, 32, 33]. Extensive tumors also present a relevant challenge to the surgical team in terms of complete tumor resection with clean margins and reconstructive procedures [18, 34].

Patients suffering from local recurrence seem to have an improved overall survival compared to a patient with regional or locoregional failure. This could be due to the still manageable tumor extent and in the case of tumor growth less aggressive tumor biology. Patients with regional recurrence or distant disease have a poor outcome.

### Therapy of recurrent disease

Taking a closer look at the chosen treatment modalities of recurrent tumors, we found a significant difference in overall survival and disease-free survival, as surgery (plus adjuvant therapy) was the favorable treatment modality. Those findings need to be interpreted in the context of pretherapeutic staging as surgically resectable localized recurrent tumors without regional or systemic spread are associated with a better outcome than tumors with extensive growth, regional or even systemic spread.

All recurrences which were classified as being not surgically curable were either referred to radio/radiochemotherapy, palliative chemotherapy, or best supportive care. As mentioned earlier, there were much more highly staged recurrent tumors referred to radiotherapy than to surgery. This must be taken into consideration when interpreting the results. A better way to figure out which modality works best would be an RCT, which to our knowledge does not exist. In addition, the inhomogeneous collective needs to be emphasized, which contains both patients treated according to guidelines and patients who had refused an indicated adjuvant treatment.

Surgery still seems to be the therapy of choice when it comes to the treatment of recurrent OSCC [18, 19, 34, 35]. Surgery alone or in combination with radiotherapy has been shown to be the most effective. When treating radiation-naïve patients with recurrent OSCC with combined surgery and radiotherapy, survival rates can be achieved that are comparable to nonrecurrent advanced OSCC [36]. Due to toxicity, radiation is limited in terms of re-radiotherapy. Surgery, on the other hand, enables several promising treatment approaches [34, 35].

Based on the assumption that inoperable or very advanced cases tend to be treated with radiotherapy or chemotherapy, our analysis shows that the treatment of recurrence should be surgically based whenever possible. In the presence of histopathological risk factors and a short disease-free interval, an adjuvant treatment should be amended. The retrospective study design has several disadvantages. The observations are certainly limited and should be interpreted carefully according to their origin. Randomized controlled trials are extremely important but also complicated to implement due to ethical concerns. Because of this, few studies can be found that have adequately achieved this [37].

Since head and neck cancer is one of the world’s most common malignant tumor entities and recurrence rates are still high, there is a need for further risk assessment in affected patients and to identify parameters that are suitable to support decision-making regarding therapy regimen for patients with recurrent OSCC [38, 39]. Based on our results, there is a sustained need for including the chronological progress and the dynamics of the tumor disease in this process. We found that patients with a disease-free interval of 12 months or less should be treated with the most powerful treatment modalities available. This means that if surgery is possible, it should strongly be considered to include adjuvant radiation or chemoradiation even when histopathological staging does not lead to this decision.

## Conclusion

Patients who suffer from tumor recurrence have a poor life expectancy. The treatment of such patients is still a great challenge for an interdisciplinary team. Based on the assumption that inoperable or very advanced cases tend to be treated with radiotherapy or chemotherapy, our analysis shows that the treatment of recurrent tumors should be surgically based and, in the presence of histopathological risk factors and short disease-free interval, should be complemented with appropriate adjuvant therapy. Provided surgical excision of the recurrence can be performed, a better outcome for patients can be expected.
